# Omega-3 supplementation from pregnancy to postpartum to prevent depressive symptoms: a randomized placebo-controlled trial

**DOI:** 10.1186/s12884-017-1365-x

**Published:** 2017-06-09

**Authors:** Juliana dos Santos Vaz, Dayana Rodrigues Farias, Amanda Rodrigues Amorim Adegboye, Antonio Egidio Nardi, Gilberto Kac

**Affiliations:** 1 Faculty of Nutrition, Pelotas Federal University, Rua Gomes Carneiro 1 – Campus Porto, Pelotas, RS 96160-000 Brazil; 2 Institute of Nutrition, Nutritional Epidemiology Observatory, Rio de Janeiro Federal University, Avenida Carlos Chagas Filho, 367, CCS – Bloco J – 2° andar, sala 29, Cidade Universitária – Ilha do Fundão, Rio de Janeiro, RJ 21941-590 Brazil; 3 Faculty of Science and Technology, University of Westminster, 115 New Cavendish Street, London, W1W 6UW UK; 4 Laboratory of Panic and Respiration, Institute of Psychiatry, Rio de Janeiro Federal University, Avenida Venceslau Braz, 71 - Botafogo, Rio de Janeiro, RJ 22290-140 Brazil

**Keywords:** Depression; pregnancy, Fatty acids, Omega-3, Randomized controlled trial

## Abstract

**Background:**

Low n-3 polyunsaturated fatty acids (PUFAs) has been linked to depression, but the preventive effect of n-3PUFAs supplementation on maternal depression needs further investigation. We aimed to evaluate the efficacy of a daily dose of n-3 PUFAs supplementation (fish oil) on the prevention of postpartum depression (PPD).

**Methods:**

A randomized, placebo-controlled, double blind trial was designed and nested into a cohort study conducted in Rio de Janeiro, Brazil. Sixty pregnant women identified as being at risk for PPD were invited and randomly assigned to receive fish oil capsules [1.8 g (1.08 g of Eicosapentaenoic (EPA) and 0.72 g of Docosapentaenoic (DHA) acids)] or placebo (control). The Edinburgh Postnatal Depression Scale (EPDS) was scored at 5–13 (T0, baseline), 22–24 (T1), 30–32 weeks of gestation (T2) and 4–6 weeks’ postpartum (T3). Supplementation started at week 22–24 of gestation (T1) and lasted for 16 weeks. Serum fatty acids were assayed to evaluate compliance. Prevalence of EPDS ≥11 was the primary outcome, and mean and changes in EPDS score, length of gestation, and birth weight the secondary outcomes. Linear mixed-effect (LME) and random-intercept logistic regression models were performed to test the effect of fish oil supplementation on prevalence of EPDS ≥11 and EPDS scores variation.

**Results:**

In intention-to-treat (ITT) analysis, at 30–32 weeks’ gestation women in the fish oil presented higher serum concentration of EPA, DHA and lower n-6/n-3 ratio comparing to the control group. There were no differences between intervention and control groups in the prevalence of EPDS ≥11, EPDS scores over time, or in changes in EPDS scores from pregnancy to postpartum in either the ITT or per-protocol analyses. Women in the fish oil group with previous history of depression presented a higher reduction on the EPDS score from the second to the third trimester in the fish oil comparing to the control group in the ITT analyses [−1.0 (−3.0–0.0) vs. -0.0 (−1.0–3.0), *P* = 0.038). These results were confirmed on the LME model (β = −3.441; 95%CI: -6.532– -0.350, *P* = 0.029).

**Conclusion:**

Daily supplementation of 1.8 g of n-3 PUFAs during 16 weeks did not prevent maternal depressive symptoms in a sample of Brazilian women.

**Trial registration:**

ClinicalTrials.gov Identifier: NCT01660165. Retrospectively registered on 24 May 2012.

## Background

The increase number of clinical and epidemiological studies showing the association between low intake of fish and seafood and increased risk of depression and mental disorders suggests that n-3 polyunsaturated fatty acids (PUFAs) might be a relevant element to treat maternal depression [1, 2]. N-3 PUFAs are essential components to human diet which must be obtained from dietary sources, as mammals are not able to synthetize de novo [3]. In addition, there is an extensive competition between n-3 and n-6 fatty acids for endogenous enzymes that limits the conversion of the n-3 precursor alpha-linoleic acid (ALA; 18:3 n-3) into neuro-active of long chain forms, specially eicosapentaenoic (EPA; 20:5 n-3) and docosahexaenoic (DHA; 22:6 n-3) [4, 5].

Pregnancy is a period of additional demands of n-3 long chain fatty acids due to high maternal transference of EPA (20:5 n-3) and DHA (22:6 n-3) to the fetus for brain growth and subsequent cognitive development [6]. Selective transportation of these fatty acids across the placenta not only biomagnifies concentrations to the fetus but may also lead to maternal depletion when dietary intake is insufficient [7, 8], which can be considered a risk factor for depression onset [9, 10].

Maternal depression is recognized as a worldwide public health issue [11]. In high-income countries, 10–20% women experience depression either during pregnancy or during the first year after birth [12–15]. In low- and lower-middle-income countries the prevalence of depression is higher and ranges from 13% to 30% in pregnancy and 13.8% to 32.9% in the postnatal period [16]. In Brazil the prevalence of depressive symptoms in the postpartum period among women living in metropolitan areas varies from 20.7% to 39.4% [17, 18].

Recent epidemiological studies conducted among Brazilian pregnant women have shown that maternal depression is associated with an unbalanced dietary intake of n-6 and n-3 fatty acids [19] and higher levels of n-6 serum concentrations [20]. Furthermore, lower serum levels of n-3 PUFAs at early pregnancy are associated with reduced fish consumption and lower inter-partum interval [21] and more anxiety symptoms [22]. A number of studies have investigated the effect of fish oil supplementation and depressive symptoms, with conflicting results [23–25]. Findings have not been consistent, neither with respect to major depression in the general populations [26] nor in pregnant and postpartum women [23–25]. Systematic reviews of these interventions also showed mixed results [26–30]. These systematic reviews were challenged by the differences regarding dose and duration of the intervention, type of population, and use of different scales to measure depression across studies, which made it difficult to estimate a pooled effect size from these studies. Therefore, the investigation of the effect of n-3 on postpartum depression in further clinical trials, designed in such way that allows comparison with previous trials, is still needed.

The use of fish oil as an alternative treatment for maternal mood disorders deserves special consideration due to potential lack of side effects for fetal development during pregnancy and breastfeeding in addition to potential neurodevelopment benefits [31]. Furthermore, limited access to health care service in low- and lower-middle-income countries makes urge to identify alternative forms of prevention and treatment for this condition, such as omega-3 supplementation [11]. Thus, this study aimed to evaluate the efficacy of a daily dose of n-3 PUFAs supplementation (fish oil) starting at second trimester of pregnancy and lasting for 16 weeks on the prevention of postpartum depression (PPD) using a randomized, placebo-controlled, double-blind design. We hypothesized that early supplementation of DHA and EPA acts as a protection against n-3 deficiency and may prevent PPD symptoms in women at high-risk.

## Methods

### Participants and setting

Study participants were recruited from a prospective observational cohort of pregnant women conducted at a public health care center in the city of Rio de Janeiro, Brazil. Enrollment was open from November 2009 to October 2011 and the last follow-up visit occurred in July 2012. The cohort eligibility criteria were defined as follows: (i) being between 5 and 13 weeks of gestation at the time of enrolment; (ii) aged between 20 and 40 years; (iii) be free from any known chronic diseases (other than obesity) such as hypertension and diabetes; (iv) residing in the study catchment area; and (v) intending to continue prenatal care in the public health center.

At the first prenatal visit, a researcher invited women to participate in the cohort study. Ninety percent of eligible women agreed to participate and 299 women were enrolled in the cohort. The cohort study included four waves: at first (T0, baseline = 5–13 weeks), second (T1 = 22–24 weeks) and third trimesters (T2 = 30–32 weeks), and postpartum (T3 = 4–6 weeks’ postpartum). Baseline visit consisted of blood collection followed by a psychiatric interview using the Mini International Neuropsychiatric Interview (MINI; version 5.0.0) [32], general questionnaire (socioeconomic data, obstetric history, lifestyle), mental health scales (including the Edinburgh Postnatal Depression Scale - EPDS), dietary intake (food frequency questionnaire, FFQ), and anthropometric assessment (weight and height). All interviews were conducted on the same day of the routine prenatal appointment at the health care center. More details on data collection for the cohort are published elsewhere [33].

### Trial inclusion and exclusion criteria

After cohort baseline data collection those women who reported a past history of depression or presented a depressive symptom score at baseline ≥9 based on the EPDS were considered as being at risk for PPD and potential candidates for the randomized clinical trial (RCT). Women that were depressed or presented psychotic symptoms, or had past history of mania or hipomania, were at suicidal risk, taking any psychiatric medication (as anti-depressive and anxiolytic) or being seen by a psychologist or psychiatrist were excluded. Furthermore, we also considered the exclusion of women taking any oil supplementation (as fish oil, flaxseed oil or cod liver oil) and those with fish or seafood allergy. However, no such cases were identified.

### Trial design and randomization

The design comprised a RCT nested to the observational cohort. Eligible women identified after the cohort baseline were randomly assigned to receive either fish oil or placebo capsules. The supplementation started after the second follow-up visit (T1) and lasted for 16 weeks. The randomization was performed by a researcher not involved in the data collection using the participant identification (subject ID) after stratification for EPDS score and previous history of depression. Participants and all research assistants and technicians responsible for running both the cohort study and the RCT were blinded to the study allocation.

### Supplementation and compliance

Participants in the RCT received 6 gelatin capsules (1 g each) per day containing either fish oil (intervention) or soybean oil (control) for 16 consecutive weeks. The capsules were identical regarding color, shape, size and packing. The fish oil capsules contained a total dose of 1.8 g of n-3 per day (1.08 g of EPA and 0.72 g of DHA). Both capsules were deodorized, and contained 0.2 mg/g of vitamin E as antioxidant. The fish oil supplement and its placebo were manufactured by Galena Nutrition® Química Industrial, São Paulo, Brazil.

Women received individual packages containing the daily dose of supplementation and were advised to take three capsules at lunch and three capsules at dinner. Within the first week, a research assistant contacted participants from both groups by phone to verify compliance and possible side effects using a standardized questionnaire. Supply of capsules was provided at week-2, week-8 (3rd trimester of follow-up visit), and week-12. Week-2 and week-12 appointments were schedule solely to provide batch of supplements and assess compliance. All participants consumed 400 μg/d of folic acid from the beginning of pregnancy, and 60 mg/d ferrous sulphate from the 2nd trimester until delivery, as provided during standard prenatal care in Brazil. Participants were asked to not alter their usual dietary habits and not consume any supplements other then the ones provided by the research team and prenatal care service.

To assess compliance, women were advised to return all empty supplement packages every visit, and a research assistant estimated the number of capsules women had taken during the interval. Additionally, serum fatty acid composition analysis was assayed as a biological marker of compliance.

### Depressive symptom measurements

The first cohort follow-up (T1) was considered baseline to the RCT. At this time point, the EPDS scale was administered and repeated after 8 weeks (at third trimester visit – T2) and 4–6 weeks’ postpartum (T3 - end-point). This scale has been previously validated for use in pregnancy [34]. Despite EPDS being considered as a screening tool rather than a diagnostic instrument for depression, this scale is widely used and it enables comparison with previous trials [23–25, 35]. The Portuguese version of the EPDS was validated in a sample of mothers from Pelotas, southern Brazil [36]. According to the validation study, the cutoff of ≥9 presents 91.3% of sensitivity and 54.7% of specificity for detecting depression compared to a semi-structured interview based on the International Statistical Classification of Disease and Related Health Problems (10th Revision) administered by mental health care professionals (gold standard) [37]. At the study end-point, for screening of moderate and severe cases of PPD, the cutoff point of ≥11 (83.8% of sensitivity and 74.7% of specificity) [36] was used.

Psychiatric interviews were conducted at T0 using the MINI [32]. This instrument consist of a standard model of a brief (15–30 min) structured interview for the evaluation of the existence of Axis I psychiatric disorders according to the Diagnostic and Statistical Manual of Mental Disorders [38]. The interview is divided into 16 modules (A-P), each one containing questions that represent different psychiatric disorders. The patients must answer ‘yes’ or ‘no’ to each of the questions; at the end of each module, and the diagnostic (yes/no) is provided. The interviews were performed by physicians and postgraduate students trained for this purpose.

### Blood sample

Fasting blood samples (5 mL) were collected by a trained technician throughout gestation (T0, T1 and T2). Serum concentrations of EPA and DHA were used to assess compliance to the fish oil supplementation, as both fatty acids measurements are used as biological markers for dietary intake [39]. The serum fatty acids composition was determined using a high-throughput robotic direct methylation method coupled with fast gas-liquid chromatography. The method was developed and validated by the Section of Nutritional Neurosciences, Laboratory of Membrane Biochemistry and Biophysics of the National Institute of Health (NIH, Bethesda, MD, USA), where the samples were analyzed [40, 41].

### Covariates

A standardized and pre-piloted questionnaire was applied at the cohort baseline to collect the following information: age (years), schooling (years), marital status (married or stable partnership/single), smoking habit (no/yes), history of depression (yes/no), alcohol consumption (no/yes), monthly per-capita family income, and parity (number of parturition).

The early pre-pregnancy body mass index (BMI, kg/m^2^) was determined from self-reported pre-pregnancy weight (kg), and height (m) measured at baseline (Seca Ltd., Hamburg, Germany). Anthropometric measurements were collected by trained interviewers according to standardized procedures [42].

A validated semi-quantitative FFQ consisting of 81 food items was administered at baseline (at 1st trimester - referring to prior 6 months of habitual diet) and at the third trimester of pregnancy (referring to dietary habits from second to third trimester) [43, 44]. At baseline, predominant dietary sources of long-chain n-3 PUFAs identified were fresh fish, canned tuna/sardines, eggs and red meat [45].

Gestational age was initially determined based on the reported date of the last menstrual period, and lately confirmed with the first ultrasound performed before 24 weeks of gestation. Obstetric outcomes such as length of gestation and birth weight were extracted from obstetric records.

### Sample size

The original sample size calculation was based on a prevalence of postpartum depression ranging from 26.9 to 39.9% according the previous studies conducted in similar population [17, 46]. We designed this study to detect a 5% reduction in the prevalence of EPDS ≥11 from baseline of 27% to 22% after the study intervention, compared with controls. The required sample size ranged from 22 to 43 for each treatment group (fish oil and control). Assuming an attrition rate of 15% due to dropouts and gestational complications, 25 to 51 participants were needed in each treatment group.

### Statistical analyses

The prevalence of depressive symptoms (EPDS score ≥ 11) was the primary outcome, while the mean and absolute changes in EPDS score and length of gestation, and birth weight were the secondary outcomes.

The data distribution was analyzed according to the Shapiro-Wilk test. Socio-economic, demographic, nutritional and gestational outcome characteristics of women in both fish oil and control groups were described using means and standard deviations or medians and interquartile ranges for symmetric and asymmetric variables, respectively. Normal distributed variables were analyzed using Student t-test or qui-square test and asymmetric variables were analyzed using Mann-Whitney U test.

We compared the serum fatty acid composition between fish oil and control groups at T0 and T1, and tested the compliance with fish oil supplementation at T2. Comparisons were performed using Mann Whitney U test.

The prevalence of depressive symptoms and mean score of EPDS were compared at T0, T1, T2 and T3 between fish oil and control groups using Mann-Whitney U test, Fisher exact test or chi-square. The absolute change (Δ) in EPDS score between end-point (T3) and T0 (cohort baseline) and between end-point (T3) and T1 (trial baseline) were tested using Mann-Whitney U test.

We performed two different longitudinal regression procedures. First, a random-intercept and slope logistic regression model having longitudinal changes in depressive symptoms (EPDS score ≥ 11/<11) as the outcome and the intervention groups as the main exposure was performed. Secondly, linear mixed-effect (LME) regression models were used to evaluate the effect of fish oil supplementation on EPDS continuous score change during pregnancy and postpartum. In the LME models, the time (weeks elapsed after conception) was included in all the models as random effects to adjust for the overall and individual variations in the EPDS score over time.

The longitudinal regression **model 1** included interactions terms between groups of intervention (fish oil vs. placebo) and time [random-intercept logistic regression = 2nd trimester (reference category) vs. 3rd trimester or postpartum; LME = continuous weeks elapsed after conception] in order to evaluate if the EPDS scores (continuous or categorical) change over time were different depending on the supplement group. Results from **model 2** were further adjusted for the following confounders: parity, education, skin color and early pre-pregnancy BMI.

All analyses were performed using both the pre-protocol and intention-to-treat (ITT) procedures. The ITT population included all women randomized, regardless of whether or not they dropped out or fully adhered to the intervention, while the per-protocol population included all women who completed the trial at each time point.

The analyses described above were repeated to compare results considering a subgroup composed of women with previous history of depression.

Statistical analyses were performed using STATA version 12.0 (Stata Corp., College Station, Texas, USA). Values were considered statistically significant when the *p*-value was lower than 0.05.

## Results

### Flowchart of participants

**Fig. 1:**
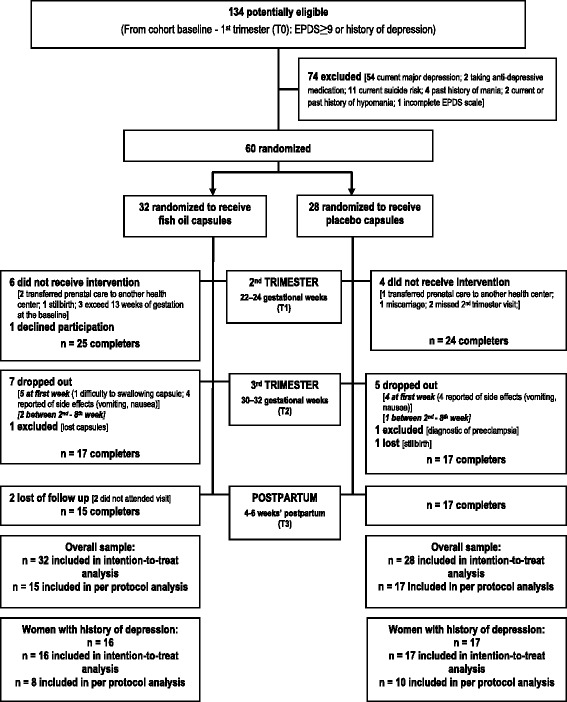
Flow of participant through the randomized clinical trial

Sixty women were randomized to receive the intervention after the second trimester data collection was performed. After randomization (second trimester visit), there was a random loss of 6 women in the fish oil (2 transferred pre-natal care to another health center, 1 reported stillbirth, 3 exceeded thirteen weeks of gestation after ultrasound was performed) and 4 in the placebo group (1 transferred pre-natal care assistant to another health center, 1 reported miscarriage and 2 missed 2nd trimester visit,) for reasons apparently not related to the study. In addition, 1 woman in the fish oil group declined participation in the RCT. Even though randomized, all these cases did not receive the intervention. After the trial commencement, 12 women dropped out the intervention (7 women from fish oil and 5 from control group), 1 woman in each intervention group was excluded (fish oil group = lost her capsules; control group = reported preeclampsia) and 1 woman in the control group had a stillbirth. At the postpartum visit, 2 women in the fish oil group missed the appointment. A total of 32 women completed the trial (Fig. 1).

### Baseline characteristics and compliance

At the baseline, there were no differences between groups, with the exception of skin color. Non-white women were more frequent in the control group (82.1%) compared with the fish oil (59.4%) (Table 1).

**Table 1:** Socio-economic, demographic and nutritional characteristics between fish oil (EPA and DHA) and control groups at the cohort baseline study (T0) (Intention-to-treat analysis)

Variable	Fish oil Supplement (*n* = 32)	Control (*n* = 28)	*p*–value
Age (years) ^a^	25.5 (22.0–34.5)	27.0 (21.0–31.0)	0.795
Gestational age (weeks) ^b^	9.7 (2.3)	9.1 (2.5)	0.336
Education (years) ^a^	11.0 (7.0–11)	8.0 (7.5–10.5)	0.070
Family income (US $) ^a^	263.2 (181.9–383.0)	304.1 (180.7–379.8)	0.811
Skin color ^c^			
White	13.0 (40.6)	5.0 (17.9)	
Non–white	19.0 (59.4)	23.0 (82.1)	0.055
Parity (number of parturition) ^c^			
0–1	26.0 (81.2)	18.0 (64.3)	
≥ 2	6.0 (18.8)	10.0 (35.7)	0.138
Early pre-pregnancy BMI (kg/m^2^) ^a^	23.9 (22.6–27.5)	23.1 (21.8–27.5)	0.213
Total dietary PUFAs (g/day) ^a^	11.3 (8.0–14.9)	12.1 (9.4–17.0)	0.334
Edinburgh Postnatal Depression Scale (Score) ^a^	10.0 (9.0–13.0)	10.5 (9.5–13.5)	0.621
History of depression ^c^			
No	16.0 (50.0)	11.0 (39.3)	
Yes	16.0 (50.0)	17.0 (60.7)	0.405

*BMI* Body mass index, *PUFAs* polyunsaturated fatty acids.

^a^Median (interquartile range); *p*-value refers to the Mann-Whitney U test.

^b^Mean (standard deviation); *p*-value refers to the Student *t* test.

^c^Absolute frequency (%); *p*-value refers to the qui-square test.

Overall, capsules were well accepted by pregnant women. Eight women (4 at the fish oil and 4 at the control group) reported nausea or vomiting, and 1 reported difficulty to swallowing the capsules. No other adverse effect was reported. Compliance was not different between groups among women who have received the intervention, with an average of 74.4 ± 24.5% and 73.5 ± 20.9% of capsules offered being consumed by the supplemented and control group, respectively (*p* = 0.903; *t* = 0.123). We also did not find statistically significant differences in compliance between fish oil and control groups when only women who have completed the trial were considered (82.4 ± 15.6% vs. 79.1 ± 16.9%; *p* = 0.559, *t* = 0.509, respectively) (Results not shown).

After eight weeks of supplementation (T2) women in the fish oil group presented higher serum concentration of total n-3, EPA, DHA and lower n-6/n-3 ratio comparing to the control group (Table 2).

**Table 2:** Longitudinal variation of median serum fatty acids during pregnancy between fish oil (EPA and DHA) and control groups (T0, T1 and T2). (Intention-to-treat analysis)

Fatty acids (μg/mL)	Fish oil Supplement (*n* = 32)	Control (*n* = 28)	*p*-value^a^
Total n-6			
T0	1018.3 (856.8–1159.9)	939.7 (793.2–1047.7)	0.076
T1	1274.8 (1098.0–1500.6)	1252.9 (1109.3–1404.2)	0.563
T2	1356.1 (1123.3–1551.3)	1362.1 (1212.6–1553.9)	0.678
Total n-3			
T0	94.7 (78.4–110.6)	95.1 (83.1–104.9)	0.976
T1	112.6 (95.0–127.3)	116.2 (105.0–132.2)	0.524
T2	154.7 (109.6–194.2)	121.1 (94.5–131.9)	0.020
n-6/n-3 ratio			
T0	11.4 (10.3–12.2)	10.3 (8.9–11.7)	0.108
T1	11.7 (9.9–13.0)	10.3 (9.7–12.4)	0.328
T2	9.5 (7.0–11.9)	11.6 (10.0–12.6)	0.004
Eicosapentaenoic acid			
T0	9.2 (7.0–11.2)	9.5 (6.5–12.3)	0.903
T1	8.7 (6.8–10.7)	8.5 (6.7–12.8)	0.813
T2	11.9 (7.6–26.4)	7.5 (4.3–11.0)	0.005
Docosahexaenoic acid			
T0	56.8 (47.1–67.4)	57.4 (48.9–68.3)	0.844
T1	67.5 (61.6–81.0)	75.5 (59.8–84.6)	0.505
T2	97.1 (66.0–120.0)	73.5 (62.8–83.9)	0.021

Evaluation was conducted at: T0 = 5–13 gestational weeks (cohort baseline); T1 = 22–24 gestational weeks (randomized clinical trial baseline); T2 = 30–32 gestational weeks.

^a^p-value refers to Mann Whitney U test; *EPA* Eicosapentaenoicacid, *DHA* Docosahexaenoicacid

### Depressive symptoms and secondary outcomes

The prevalence of EPDS ≥11 did not differ significantly between fish oil and control at any time in pregnancy or postpartum for neither the ITT or per protocol analysis, nor even when analysis considered only those with previous history of depression (Table 3).

**Table 3:** Depressive symptoms (EPDS score ≥ 11) during pregnancy and postpartum between fish oil (EPA and DHA) and control groups (T0, T1, T2, and T3)

		T0			T1			T2			T3		
	N (%)	Depressive symptoms		*p* ^a^	Depressive symptoms		*p* ^a^	Depressive symptoms		*p* ^a^	Depressive symptoms		*p* ^a^
		n	Prevalence		n	Prevalence		n	Prevalence		n	Prevalence	
Overall sample													
Intention-to-treat													
Control	28 (46.7)	14	50.0		7	25.0		8	28.6		7	25.0	
Fish oil	32 (53.3)	15	46.9	0.809	12	37.5	0.299	11	34.4	0.630	8	25.0	1.000
Per protocol													
Control	17 (53.1)	9	52.9		4	23.5		5	29.4		3	17.6	
Fish oil	15 (46.9)	9	60.0	0.688	8	53.3	0.082	7	46.7	0.314	5	33.3	0.306
Women with previous history of depression													
Intention-to-treat													
Control	17 (51.5)	9	52.9		5	29.4		7	41.2		7	41.2	
Fish oil	16 (48.5)	6	37.5	0.373	7	43.8	0.392	7	43.8	0.579	5	31.3	0.554
Per protocol													
Control	10 (55.6)	5	50.0		3	30.0		4	40.0		3	30.0	
Fish oil	8 (44.4)	4	50.0	1.000	5	62.5	0.168	4	50.0	0.671	3	37.5	0.737

Evaluation was conducted at: T0 = 5–13 gestational weeks (cohort baseline); T1 = 22–24 gestational weeks (randomized clinical trial baseline); T2 = 30–32 gestational weeks; T3 = 4–6 weeks’ postpartum.

*EPDS* Edinburgh Postnatal Depression Scale, *EPA* Eicosapentaenoic acid, *DHA* Docosahexaenoic acid.

^a^p-value refers to chi-square test or Fisher exact test.

There were no differences between fish oil and control group in the EPDS scores over time, as well as change in EPDS score from pregnancy to postpartum for neither the ITT or per protocol analysis. It was observed a higher reduction on the EPDS score from the second to the third trimester in the fish oil comparing to the control group, in the ITT analyses [−1.0 (−3.0–0.0) vs. -0.0 (−1.0–3.0), *P* = 0.038) (Table 4).

**Table 4:** Longitudinal variation of EPDS score during pregnancy and postpartum between fish oil (EPA and DHA) and control groups

	EPDS score				Absolute change in EPDS score	
	T0	T1	T2	T3	Δ (T3 – T1)	Δ (T3 – T0)
Overall sample						
Intention-to-treat						
Control (*n* = 28)	10.5 (9.5–13.5)	8.5 (6.0–10.5)	7.0 (5.5–11.0)	6.0 (5.0–11.0)	−1.0 (−3.5–1.5)	−4.0 (−8.0–0.0)
Fish oil (*n* = 32)	10.0 (9.0–13.0)	9.0 (5.5–13.0)	6.5 (4.0–12.0)	6.5 (3.5–10.5)	−1.0 (−4.0–0.0)	−4.0 (−6.5–0.0)
p-value^1^	0.612	0.721	0.607	0.612	0.512	0.748
Per protocol						
Control (*n* = 17)	11.0 (9.0–15.0)	8.0 (6.0–10.0)	7.0 (5.0–11.0)	6.0 (5.0–7.0)	−1.0 (−4.0–1.0)	−5.0 (−8.0 – −3.0)
Fish oil (*n* = 15)	11.0 (10.0–14.0)	11.0 (5.0–14.0)	10.0 (4.0–15.0)	8.0 (5.0–12.0)	−1.0 (−5.0–1.0)	−4.0 (−8.0–0.0)
p-value^1^	0.675	0.178	0.635	0.216	0.955	0.404
Women with previous history of depression						
Intention-to-treat						
Control (*n* = 17)	11.0 (10.0–15.0)	9.0 (5.0–12.0)	7.5 (5.5–11.0)	7.0 (5.0–14.0)	0.0 (−1.0–3.0)	−3 .0 (−8.0–0.0)
Fish oil (*n* = 16)	10.0 (7.0–12.5)	8.5 (5.5–13.5)	9.0 (5.0–15.0)	7.0 (3.0–11.0)	−1.0 (−3.0–0.0)	−1.0 (−6.0–0.0)
p-value^1^	0.318	0.527	0.709	0.322	0.038	0.201
Per protocol						
Control (*n* = 10)	11.0 (8.0–15.0)	7.5 (5.0–12.0)	7.5 (5.0–11.0)	6.5 (5.0–12.0)	−0.5 (−2.0–2.0)	−4.5 (−8.0–0.0)
Fish oil (*n* = 8)	10.5 (10.0–15.0)	13.0 (6.0–14.5)	12.5 (5.0–15.5)	8.5 (6.5–12.0)	−1.5 (−4.0–0.5)	−4.5 (−7.0–1.0)
p-value^1^	0.893	0.117	0.303	0.421	0.264	0.561

Evaluation was conducted at: T0 = 5–13 gestational weeks (cohort baseline); T1 = 22–24 gestational weeks (randomized clinical trial baseline); T2 = 30–32 gestational weeks; T3 = 4–6 weeks’ postpartum.

Data are presented as median (interquartile range), ^*1*^p–value refers to Mann Whitney U test.

*EPDS* Edinburgh Postnatal Depression Scale, *EPA* Eicosapentaenoic acid, *DHA* Docosahexaenoic acid.

**Fig. 2:**
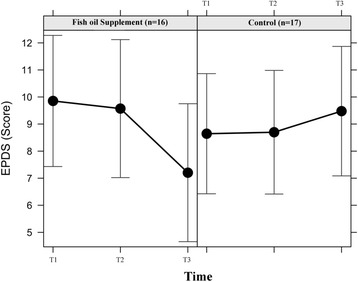
Longitudinal changes in EDPS score in women with previous history of depression according to intervention group (Intention-to-treat analysis). Note: T1 = 22–24 gestational weeks (Randomized Clinical Trial baseline); T2 = 30–32 gestational weeks; T3 = 4–6 weeks’ postpartum. Data are presented as linear mixed effect coefficient (β) and 95% CI. *P*-value refers to the maximum likelihood estimator. T1 was the reference category. Fish oil supplement group: β_(T2)_ = −0.266; 95% CI = −2.428 to 1.896; *P* = 0.809. β_(T3)_ = −2.665; 95% CI = −4.774 to −0.556; *P* = 0.013. Control group: β_(T2)_ = 0.046; 95% CI = −2.118 to 2.210; *P* = 0.967. β_(T3)_ = 0.891; 95% CI = −1.364 to 3.146; *P* = 0.439

A sharper decline was observed in EPDS score from the second trimester to postpartum for women who reported previous history of depression before pregnancy in the fish oil when compared to the control group (ß = −2.665, 95% CI: -4.774 – -0.556) (Fig. 2).

No effect of the intervention on EPDS ≥11 was found, neither on the ITT or per-protocol analysis, or when the analyses were performed for those with previous history of depression (Table 5).

**Table 5:** Random-intercept longitudinal logistic regression of fish oil (EPA and DHA) supplementation on depressive symptoms (EPDS score ≥ 11) during pregnancy^a^ and postpartum^b^

	Model 1^c^	*p* ^f^	Model 2^d^	*p* ^f^
	OR^e^ (95% CI)		β^e^ (95% CI)	
Overall sample				
Intention-to-treat				
Supplement (Control vs. Fish oil)	3.338 (0.347–32.122)	0.297	3.376 (0.220–51.757)	0.382
Time				
T1 vs. T2	0.848 (0.092–7.776)	0.884	1.566 (0.195–12.572)	0.673
T1 vs. T3	0.119 (0.001–10.666)	0.353	0.722 (0.037–14.110)	0.830
Interaction terms Time # Supplement				
T2 # Fish oil	0.375 (0.023–6.094)	0.491	0.334 (0.023–4.871)	0.423
T3 # Fish oil	0.112 (0.001–8.946)	0.328	0.137 (0.005–3.824)	0.242
Women with previous history of depression				
Intention-to-treat				
Supplement (Control vs. Fish oil)	2.812 (0.183–43.201)	0.458	2.833 (0.223–36.012)	0.422
Time				
T1 vs. T2	2.358 (0.188–29.622)	0.506	2.404 (0.240–24.095)	0.456
T1 vs. T3	1.878 (0.066–53.242)	0.712	1.848 (0.084–40.854)	0.697
Interaction terms Time # Supplement				
T2 # Fish oil	0.237 (0.007–7.951)	0.422	0.239 (0.008–7.052)	0.407
T3 # Fish oil	0.034 (0.0001–7.597)	0.221	0.033 (0.0002–6.986)	0.213

Per-protocol analysis had a reduced sample size and were not presented due to imprecise results.

*EPDS* Edinburgh Postnatal Depression Scale, *CI* confidence interval, *EPA* Eicosapentaenoic acid, *DHA* Docosahexaenoic acid

^a^Pregnancy period comprised T1 (22–24 gestational weeks - randomized clinical trial baseline) and T2 (30–32 gestational weeks);

^b^Postpartum period comprised T3 (between 4 and 6 weeks’ postpartum);

^c^Model 1 was adjusted for the interaction between time (T1 - T3) and fish oil supplement;

^d^Model 2 was additionally adjusted for gestational age, parity, education, skin color and early pre-pregnancy BMI;

^e^OR **=** Logistic Mixed Effect odds ratio

^f^p-value refers to maximum likelihood estimator.

No effect of the intervention on EPDS scores was found between T1 and T2 and T1 and T3 for ITT when intervention and the control group were compared. Similar results were observed for the per-protocol analysis. We found a significant reduction from T1 to T3 in the ITT analysis when only women with previous history of depression were considered (β = −3.441; 95% CI: -6.532 – -0.350, *P* = 0.029) (Table 6).

**Table 6:** Longitudinal effect of fish oil (EPA and DHA) supplementation on EPDS score during pregnancy^a^ and postpartum^b^

	Model 1^c^	*p* ^f^	Model 2^d^	*p* ^f^
	β^e^ (95% CI)		β^e^ (95% CI)	
Overall sample				
Intention-to-treat				
Supplement (Control vs. Fish oil)	0.597 (−1.771–2.965)	0.621	0.761 (−1.678–3.201)	0.541
Time				
T1 vs. T2	−0.480 (−2.166–1.157)	0.566	−0.481 (−2.119–1.157)	0.565
T1 vs. T3	−0.894 (−2.532–0.743)	0.284	−0.890 (−2.528–0.748)	0.287
Interaction terms Time # Supplement				
T2 # Fish oil	−0.745 (−3.061–1.570)	0.528	−0.734 (−3.051–1.583)	0.535
T3 # Fish oil	−1.647 (−3.959–0.665)	0.163	−1.651 (−3.963–0.662)	0.162
Per protocol				
Supplement (Control vs. Fish oil)	2.620 (−0.642–5.881)	0.115	2.446 (−0.859–5.752)	0.147
Time				
T1 vs. T2	−0.172 (−2.368–2.024)	0.877	−0.160 (−2.294–1.989)	0.884
T1 vs. T3	−1.189 (−3.435–1.056)	0.299	−1.172 (−3.470–1.126)	0.318
Interaction terms – Time # Supplement				
T2 # Fish oil	−0.850 (−4.058–2.357)	0.603	−0.864 (−4.003–2.276)	0.590
T3 # Fish oil	−1.189 (−4.468–2.089)	0.477	−1.260 (−4.613–2.093)	0.461
Women with previous history of depression				
Intention-to-treat				
Supplement (Control vs. Fish oil)	1.210 (2.033–4.453)	0.465	1.175 (−2.052–4.401)	0.475
Time				
T1 vs. T2	0.054 (−2.011–2.120)	0.959	0.053 (−2.008–2.114)	0.960
T1 vs. T3	0.834 (−1.242–2.910)	0.431	0.843 (−1.238–2.924)	0.427
Interaction terms Time # Supplement				
T2 # Fish oil	−0.337 (−3.471–2.796)	0.833	−0.306 (−3.432–2.820)	0.848
T3 # Fish oil	−3.484 (−6.567 – −0.401)	0.027	−3.441 (−6.532– −0.350)	0.029
Per protocol				
Supplement (Control vs. Fish oil)	3.466 (−0.497–7.429)	0.087	3.623 (−0.192–7.438)	0.063
Time				
T1 vs. T2	0.099 (−2.586–2.785)	0.942	0.101 (−2.584–2.786)	0.941
T1 vs. T3	−0.098 (−2.785–2.589)	0.943	−0.101 (−2.788–2.587)	0.941
Interaction terms – Time # Supplement				
T2 # Fish oil	−0.474 (−4.502–3.555)	0.818	−0.476 (−4.503–3.552)	0.817
T3 # Fish oil	−2.398 (−6.429–1.632)	0.244	−2.400 (−6.431–1.631)	0.243

*EPDS* Edinburgh Postnatal Depression Scale, *CI* confidence interval, *EPA* Eicosapentaenoic acid, *DHA* Docosahexaenoic acid

^a^Pregnancy period comprised T1 (22–24 gestational weeks) and T2 (30–32 gestational weeks);

^b^Postpartum period comprised T3 (between 4 and 6 weeks’ postpartum);

^c^Model 1 was adjusted for the interaction between time (T1 - T3) and fish oil supplement;

^d^Model 2 was additionally adjusted for gestational age, parity, education, skin color and early pre-pregnancy BMI;

^e^β **=** Linear Mixed Effect (LME) coefficient

^f^p–value refers to maximum likelihood estimator.

No differences in gestational length (39.0 ± 1.9 vs. 39.1 ± 1.8, *P* = 0.859) and birth weight (3322.7 ± 561.6 vs. 3210 ± 553.1, *P* = 0.456) were observed between fish oil and control groups (Results not shown).

## Discussion

Our findings showed that in a sample of pregnant women at risk for PPD and low fish intake a daily supplementation of 1.8 g of n-3 per day (1.08 g of EPA and 0.72 g of DHA) had no significant effect on mean depression scores and occurrence of major depressive symptoms during pregnancy and early postpartum. Despite significant increase in serum levels of EPA, DHA and lower n-6/n3 ratio in the third trimester these changes did not affect EPDS scores at early postpartum. These findings are in line with some earlier preventive trials [23, 35, 47, 48]. In contrast, we found that women in the fish oil group with previous history of depression presented a sharper decline in the EPDS score from the second trimester to postpartum comparing to the control group. These results were confirmed by the multiple longitudinal regression model.

One previous RCT with 138 women investigating whether 200 mg DHA/day supplementation in the first four months after delivery could prevent postpartum depression found no significant results [47]. One potential explanation might be the late initiation of the supplementation, as compared with the gradual decline of maternal DHA status during pregnancy. However, another RCT offering 220 mg DHA/day (*n* = 42) or DHA and arachidonic acid (20:4n-6; *n* = 41) (both 220 mg/day) from week 16 of gestation up to 12 weeks postpartum did not find significant results related to prevention of postpartum depressive symptoms either [35].

It has also been argued that EPA has a greater effect in preventing depression and alleviating symptoms than DHA [49]. However, a three arm trial (*n* = 42 in each arm) offering daily supplementation of 1) EPA-rich fish oil capsules (4:1 ratio EPA/DHA, 1060 mg EPA plus 274 mg DHA); 2) DHA-rich oil capsules (4:1 ratio DHA/EPA, 900 mg DHA plus 180 mg EPA), and 3) placebo from 12 to 20 weeks of gestation to 8 weeks postpartum found that neither EPA nor DHA supplementation prevented depressive symptoms [48].

It is worth discussing the placebo effect, which all RCTs are subject to. In the case of mood disorders, depressive symptoms may be masked by simply participating in a trial with increased contact with researchers or health care professionals, which creates a therapeutic environment that activates placebo benefits in both trial groups and possibly resulting in spontaneous remission of depressive symptoms [50].

Most of the observational studies that investigated depression from pregnancy to postpartum period reported a significant decrease in depressive scores over time [12, 51]. This finding is in line with those results observed in our main cohort study [33, 52, 53]. Some authors suggested that a decrease in depression may be due to family and social support and coping behavior and skills [54] specially observed among second-time mothers [55]. Thus, the possible protective effect of fish oil supplementation could be observed in a time-point later than 4–6 weeks’ postpartum.

The question that hovers over RCTs conducted during pregnancy is the definition of the supplement dose used to treat or prevent mood disorders. Specific recommendations for DHA and EPA supplementation during pregnancy have not been fully established due to insufficient data on the benefits to maternal and infant outcomes (e.g., prematurity). The recommendation of the American Dietetic Association and Dieticians of Canada is that adults, including pregnant and lactating women, should have a combined intake of 500 mg/day of DHA and EPA based on an average intake of 2000 kcal/day [56]. Following the guidance of the American Institute of Medicine [57], seafood intake during pregnancy and lactation should be approximately 200–300 mg DHA/day. The Perinatal Lipid Intake Working Group on behalf of the European Commission has reviewed all available evidence and recommended an intake of ≥200 mg DHA/day for pregnant and lactating women [58]. Since the dietary requirement for DHA and EPA for a healthy pregnancy has not been clearly established, it may be difficult to define the ideal dose for an intervention. Despite fish and seafood products being considered best sources of EPA and DHA, the intake of these food items was low in our population, i.e. 39% of women in the cohort did not report intake of fresh fish, and 34% reported intake once a month. Overall, the median dietary intake of EPA and DHA was 0.025 g (interquartile range = 0.01–0.03) per day and 0.026 g (interquartile range = 0.0–0.03) per day [45] respectively, which was far bellow the recommendations.

Although previous studies have investigated the effect of fish oil supplementation during pregnancy and postpartum on depression [10, 23, 24, 35], to the best of our knowledge this is one of the few studies with focus on prevention of postpartum depressive symptoms in women at high risk and provided longer period of supplementation covering the second trimester of pregnancy to early postpartum. The study design (double-blind) reduces the risk of the bias and increases the credibility in the results. An additional strength of this study is the use of biomarkers to evaluate intervention compliance. Our results showed that in the fish oil group serum levels of EPA and DHA significantly were higher and n-6/n-3 ratio was lower in the third trimester compared to the control group. Another important strength of this study is the use of biological samples in the investigation of mood disorders. Serum levels of fatty acids are known as biological markers of dietary intake in epidemiological investigation [39]. The serum composition of essential n-6 and n-3 fatty acids assessed in the first trimester (cohort baseline) partially reflects the usual diet with less interference of the gestational period [45]. A further strength is the use of different instruments, including a validated scale (EPDS) to measure depressive symptoms and a structured interview to assess the diagnosis of major depressive episodes (MINI). Most RCTs investigated depression based solely on scales used for screening of depression [23, 35, 47]. Although EPDS is a self-reported questionnaire, this scale is widely used in psychiatry studies with pregnant women; thus it allows comparisons and enhances the credibility of our findings, as current results were consistent across different scales and outcome measures.

Although the early initiation of the supplementation and relatively high dose of EPA compared to some previous studies, the current RCT had no effect on depressive symptoms during pregnancy and postpartum. This finding might be explained by the fact that the effect is too subtle to be detected even in large sample size RCTs [23, 35, 47, 48]. Although ITT analyses, whereby all patients randomly assigned to one of the study arms are analyzed, regardless of whether or not they completed or received the full intervention, is a rather conservative approach, findings remained unaltered when excluding women who did not receive the intervention or lost to follow up.

We found in this Brazilian RCT that women in the fish oil group with previous history of depression presented a significant reduction in the EPDS score from pregnancy to postpartum when results where compared to the control group. Our results are in line with the hypothesis-testing meta-analysis of Hallahan et al. [59] that have shown that the efficacy of omega-3 supplementation has significant beneficial on subjects with a diagnosis of depressive illness compared to placebo, with no benefit observed in non-depressed groups. Depression symptoms are difficult to be measured and changes in mood in non-clinical depressive populations might not necessarily have the same translation as in clinical depressive subjects [59]. This may justify why we only observed a significant difference in EPDS score among those women with previous history of depression, as this condition may represent recurrent cases with periods of normal function between episodes of depression.

Furthermore, higher dosages might be needed, particularly in a population with low EPA and DHA dietary intake. In this study, the maximum commercial available dose in Brazil was used; attempts to obtain capsules with higher EPA and DHA were made, however national regulations to import pharmaceutical products precluded any possibility to purchase supplements from international industries. To achieve a dose of 1.8 g of long chain n-3 (DHA + EPA) per day, women needed to take 6 capsules per day. However, compliance was satisfactory despite the fact that 8 women could not tolerate the ingestion of 6 capsules per day due to nausea and vomiting which are common symptoms during pregnancy.

## Conclusions

In a population of Brazilian pregnant women at risk to PPD and low fish intake a daily dose of 1.8 g of n-3 PUFAs (1.08 g of EPA and 0.72 g of DHA) for 16 weeks starting at 22–24 weeks of pregnancy did not prevent depressive symptoms in pregnancy and early postpartum. The possible benefit in women with history of depression must be further investigated, as only few trials have particularly focused on high-risk women and results are still conflicting.

## Abbreviations

BMI: Body Mass Index
DHA: Docosapentaenoic acid
EPA: Eicosapentaenoic acid
EPDS: Edinburgh Postnatal Depression Scale
ICD: International Classification of Disease
ID: Identification
ITT: Intention-To-Treat
LME: Linear Mixed-Effect
MINI: Mini International Neuropsychiatric Interview
PPD: Postpartum depression
PUFAs: Polyunsaturated fatty acids
RCT: Randomized Controlled Trial
T: Time
